# Reappraisal of a 2-cm Cutoff Size for the Management of Nonfunctional Pancreatic Neuroendocrine Tumors: A Population-Based Study

**DOI:** 10.3389/fendo.2022.928341

**Published:** 2022-07-18

**Authors:** Zhen Yang, Dongsheng Zhang, Guangjun Shi

**Affiliations:** Department of Hepatopancreatobiliary Surgery, Qingdao Municipal Hospital, Qingdao University, Qingdao, China

**Keywords:** pancreatic neuroendocrine tumors, observation, surgery, tumor size, survival

## Abstract

**Background:**

Expectant observation and aggressive surgery are both recommended for small nonfunctional pancreatic neuroendocrine tumors (NF-PanNETs). However, the optimal management of small NF-PanNETs remains disputable due to the heterogeneous clinical behavior.

**Methods:**

Patients who were diagnosed with pancreatic neuroendocrine neoplasms (PanNENs) between 2000 and 2018 were identified from the surveillance, epidemiology, and end results (SEER) database and reviewed retrospectively. Tumor aggressiveness was defined as poor differentiation, lymph node involvement, liver involvement, and advanced stage. The best cutoff of tumor size associated with tumor aggressiveness was determined through the receiver operating characteristic (ROC) curve analysis. Univariate and multivariate analyses were used to identify prognostic factors in patients with tumors of ≤2 cm.

**Results:**

A total of 5,172 patients with PanNENs were enrolled, including 1,760 (34.0%) tumors ≤2 cm and 3,412 (66.0%) tumors >2 cm. A 2.5-cm cutoff size was found to be associated with a satisfactory ability in predicting tumor aggressiveness. On multivariate analysis, age, gender, ethnicity, tumor grade, tumor number, and stage were independent prognostic factors for overall survival (OS) in patients with tumors less than or equal to 2 cm in size. A total of 1,621 patients were diagnosed with NF-PanNETs according to the WHO classification, of whom 1,350 underwent surgery, 271 performed active observation. The OS was significantly better in the surgery group compared to the observation group regardless of propensity score analysis. Additionally, a total of 407 patients were selected based on the multivariate Cox regression analysis, of whom 46 underwent observation, 361 underwent surgery, and the OS was comparable.

**Conclusion:**

Expectant observation may be a reasonable alternative to aggressive surgical resection in highly selected small NF-PanNET patients. Also, the decision to observe versus surgery should not only be based on tumor size alone but also take into account other important clinicopathological factors.

## Introduction

Pancreatic neuroendocrine neoplasms (PanNENs) are among the heterogeneous group of neoplasms with the most rapidly increasing incidence recently (1, 2). This increase is largely attributed to the advances in diagnostic techniques, including computed tomography and endoscopy. Unlike pancreatic adenocarcinoma, the vast majority of PanNENs are considered clinically indolent diseases and associated with favorable prognoses (3, 4). Clinically, PanNENs are classified into functional and nonfunctional diseases. Different from functional PanNENs (F-PanNENs) that are combined with syndromes of hormone hypersecretion, nonfunctional PanNENs (NF-PanNENs) are not accompanied by clinically significant hormonal symptoms. With the wide use of cross-sectional imaging, a sizable fraction of patients are incidentally diagnosed with small, asymptomatic NF-PanNENs. To date, the natural history is, however, not well described. According to the WHO classification, PanNENs are classified into well-differentiated, low-to-intermediate-grade pancreatic neuroendocrine tumors (PanNETs) and poorly differentiated, high-grade pancreatic neuroendocrine carcinomas (PanNECs) (5, 6). The management of F-PanNETs has less controversies, while the treatment of NF-PanNETs, especially for tumors less than or equal to 2 cm in size, remains disputable (7–9). Incidentally diagnosed NF-PanNETs generally exhibit benign behaviors, making them suitable and feasible to undergo surveillance according to some guidelines. In addition, radical treatments such as pancreatectomy may carry a high risk of developing postoperative complications. However, there are limited data examining the safety of this conservative policy. Also, some studies found that NF-PanNETs are inclined to have lymph node involvement, which may compromise the survival results in patients who conduct a “wait-to-see” strategy (10, 11). In terms of the potential risks and uncertain benefits of the observation strategy, the recommendation for its use should be interpreted with caution.

Therefore, the purpose of the study was to identify the association between tumor size and aggressive behaviors in NF-PanNET patients as well as to compare the long-term survival outcomes between close observational monitoring and aggressive surgical resection among patients with NF-PanNETs ≤2 cm. In addition, we attempted to identify patients who were potential candidates for an observational treatment based on a large population database from the United States.

## Patients and Methods

**Figure 1:**
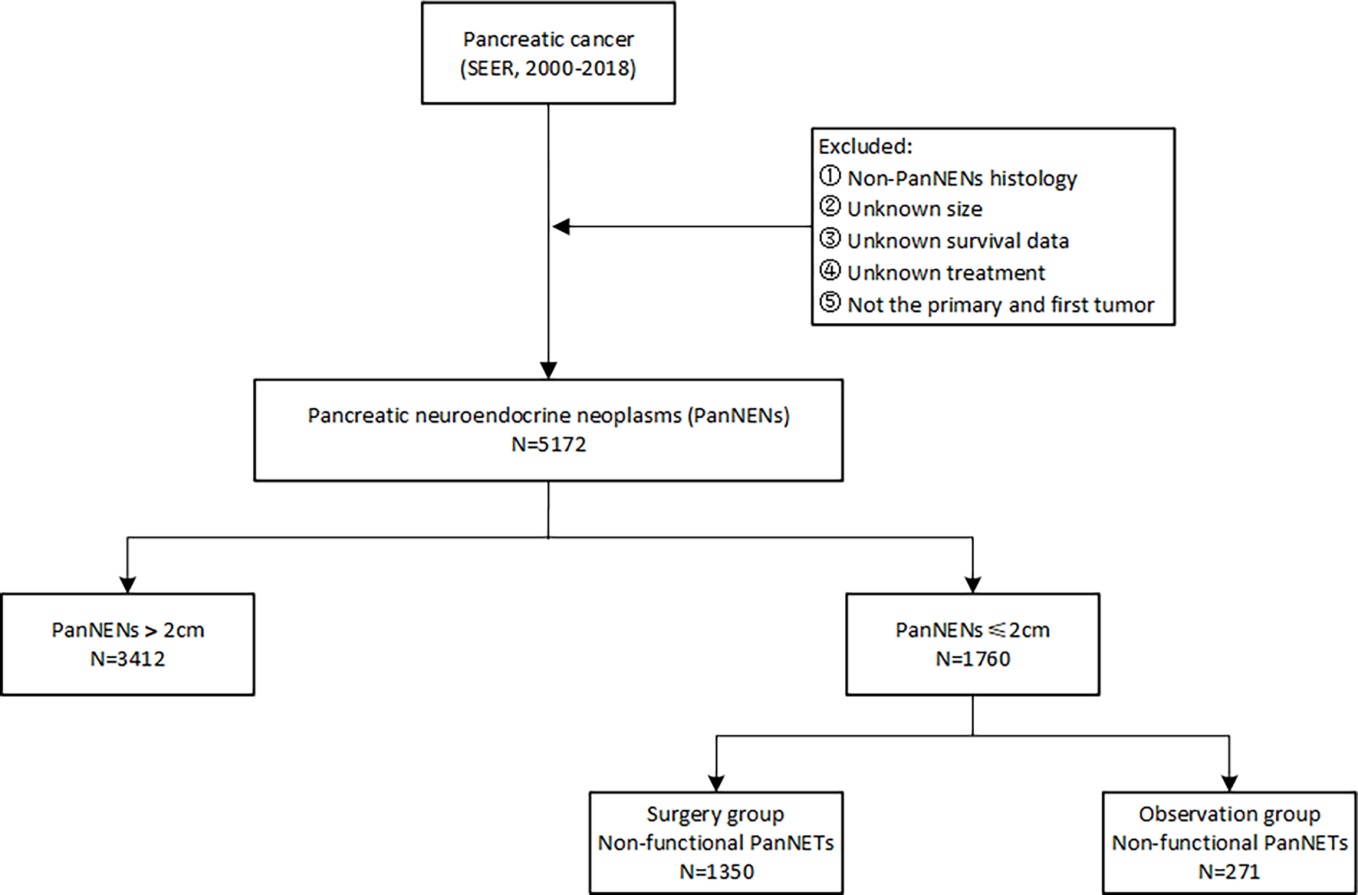
The workflow of patient selection for this study.

In this retrospective study, patients diagnosed with PanNENs between 2000 and 2018 were identified from the surveillance, epidemiology, and end results (SEER) database. The evaluated variables included age at diagnosis, gender, year of diagnosis, ethnicity, marital status, tumor characteristics, functionality, treatment, and survival outcomes. The inclusion criteria for PanNENs based on the International Classification of Diseases for Oncology, third edition (ICD-O-3) were as follows: primary sites C25.0 to C25.9 with histological codes 8150, 8151, 8152, 8153, 8155, 8156, 8240, 8241, 8242, 8243, 8245, 8246, and 8249. Patients with unknown information on vital status and survival duration were excluded. The workflow of patient selection for this study is detailed in **Figure 1**. The long-term survival outcomes were compared between the observation and surgery cohorts by evaluating the overall survival (OS) and cancer-specific survival (CSS) before and after propensity score matching.

### Statistical Analysis

All statistical analyses were performed using R software and SPSS with a two-sided significance level of 0.05. The categorical variables were presented as numbers (percentage) and were assessed between groups with the Chi-square (*χ*²) test or Fisher’s exact test, as appropriate. Continuous variables were described as mean ± standard deviation (SD) or median (interquartile range) and were compared using the Student’s *t*-test or the Mann–Whitney *U* test, as appropriate. Multivariate analysis examining who was more likely to perform surgery was conducted using predictive factors statistically significant to univariate analysis. Furthermore, Cox proportional hazard regression model was used to determine the prognostic variables in patients with tumors ≤2 cm. A propensity score matching (PSM) method using a logistic regression model was utilized to reduce the selection biases and balance confounding factors. In addition, an optimal cutoff value of tumor size for predicting tumor aggressiveness was defined as poor differentiation, lymph node involvement, liver involvement, and advanced stage using the receiver operating characteristics (ROC) method. Survival results were estimated with the Kaplan-Meier method and compared by the log-rank test between groups. In order to evaluate the efficacy of expectant observation in NF-PanNETs with less than or equal to 2 cm in size, OS and CSS rates were compared between observation and aggressive surgical resection cohorts before and after PSM.

## Results

### Baseline Characteristics

**Figure 2:**
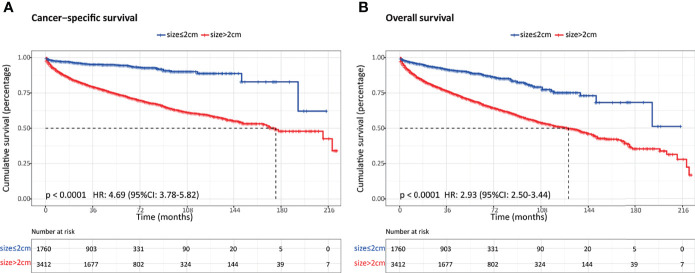
Comparison of survival outcomes between patients with pancreatic neuroendocrine neoplasms (PanNENs) ≤2 cm and PanNENs >2 cm. **(A)** Cancer-specific survival. **(B)** Overall survival.

Overall, a total of 5,172 patients with PanNENs between 2001 and 2018 were identified from the SEER database, including 1,760 (34.0%) tumors ≤2 cm and 3,412 (66.0%) tumors >2 cm. Among the cohort with PanNENs ≤2 cm, a vast majority of patients were white (76.1%), married (62.8%), younger than 65 years old (61.8%), well-differentiated (86.4%), and had the loco-regional disease at diagnosis (95.3%). Of note, about 82.7% of these neoplasms were surgically resected while only 0.7% received radiation and 2.1% received chemotherapy. As for patients with tumors larger than 2 cm, the baseline characteristics were significantly different from those with tumors ≤2 cm. Patients with PanNENs >2 cm presented with a more advanced tumor burden, including higher proportions of poor differentiation, lymph node involvement, liver involvement, and late tumor stage. Additionally, the rate of surgical treatment was significantly lower compared to that in patients with PanNENs ≤2 cm. The more detailed clinical characteristics are summarized in **Table 1**. Kaplan–Meier curves revealed that PanNENs >2 cm were associated with worse survival outcomes than PanNENs ≤2 cm (**Figure 2**).

**Table 1:** Patient characteristics of PanNENs ≤2 cm versus >2 cm in the SEER database.

Variables	PanNENs (size ≤2 cm)	PanNENs (size >2 cm)	*p*-value
Total	1,760 (34.0%)	3,412 (66.0%)	
Age			0.004^*^
<65** **years	1,087 (61.8%)	2,245 (65.8%)	
≥65** **years	673 (38.2%)	1,167 (34.2%)	
Gender			<0.001^*^
Male	845 (48.0%)	1,934 (56.7%)	
Female	915 (52.0%)	1,478 (43.3%)	
Ethnicity			0.057
White	1,339 (76.1%)	2,667 (78.2%)	
Black	203 (11.5%)	397 (11.6%)	
Other	218 (12.4%)	348 (10.2%)	
Marital status			0.970
Married	1,105 (62.8%)	2,144 (62.8%)	
Other	655 (37.2%)	1,268 (37.2%)	
Tumor grade			<0.001^*^
Well differentiated	1,521 (86.4%)	2,054 (60.2%)	
Moderately differentiated	198 (11.3%)	892 (26.1%)	
Poorly differentiated	41 (2.3%)	466 (13.7%)	
Tumor number			0.021^*^
Single	1,572 (89.3%)	3,115 (91.3%)	
Multiple	188 (10.7%)	297 (8.7%)	
Tumor location			<0.001^*^
Head	433 (24.6%)	1,110 (32.5%)	
Body/tail	1,033 (58.7%)	1,677 (49.2%)	
Other	294 (16.7%)	625 (18.3%)	
Functional status			<0.001^*^
Functional	85 (4.8%)	345 (10.1%)	
Nonfunctional	1,675 (95.2%)	3,067 (89.9%)	
Lymph node involvement			<0.001^*^
Yes	148 (8.4%)	1,245 (36.5%)	
No	1,612 (91.6%)	2,167 (63.5%)	
Liver involvement			<0.001^*^
Yes	57 (3.2%)	735 (21.5%)	
No	1,676 (95.3%)	2,315 (67.9%)	
Unknown	27 (1.5%)	362 (10.6%)	
Tumor stage			<0.001^*^
Localized	1,481 (84.1%)	1,174 (34.4%)	
Regional	196 (11.2%)	1,082 (31.7%)	
Distant	83 (4.7%)	1,156 (33.9%)	
Surgery			<0.001^*^
Yes	1,456 (82.7%)	2,482 (72.7%)	
No	304 (17.3%)	930 (27.3%)	
Radiation			<0.001^*^
Yes	13 (0.7%)	171 (5.0%)	
No	1,747 (99.3%)	3,241 (95.0%)	
Chemotherapy			<0.001^*^
Yes	37 (2.1%)	699 (20.5%)	
No	1,723 (97.9%)	2,713 (79.5%)	
Primary endpoint: OS (months)			
Mean (95% CI)	168.3 (157.5–179.0)	124.0 (119.2–128.8)	<0.001^a,*^
Median (95% CI)	NE	129.0 (116.2–141.8)	<0.001^a,*^
Primary endpoint: CSS (months)			
Mean (95% CI)	188.4 (177.3–200.0)	141.3 (136.2–146.3)	<0.001^a,*^
Median (95% CI)	NE	176.0 (149.5–184.2)	<0.001^a,*^

PanNENs, pancreatic neuroendocrine neoplasms; SEER, surveillance, epidemiology, and end results; OS, overall survival; CI, confidence interval. ^a^Log-rank test. ^*^Significance.

### Predictor of Aggressive Behavior: Role of Tumor Size

**Figure 3:**
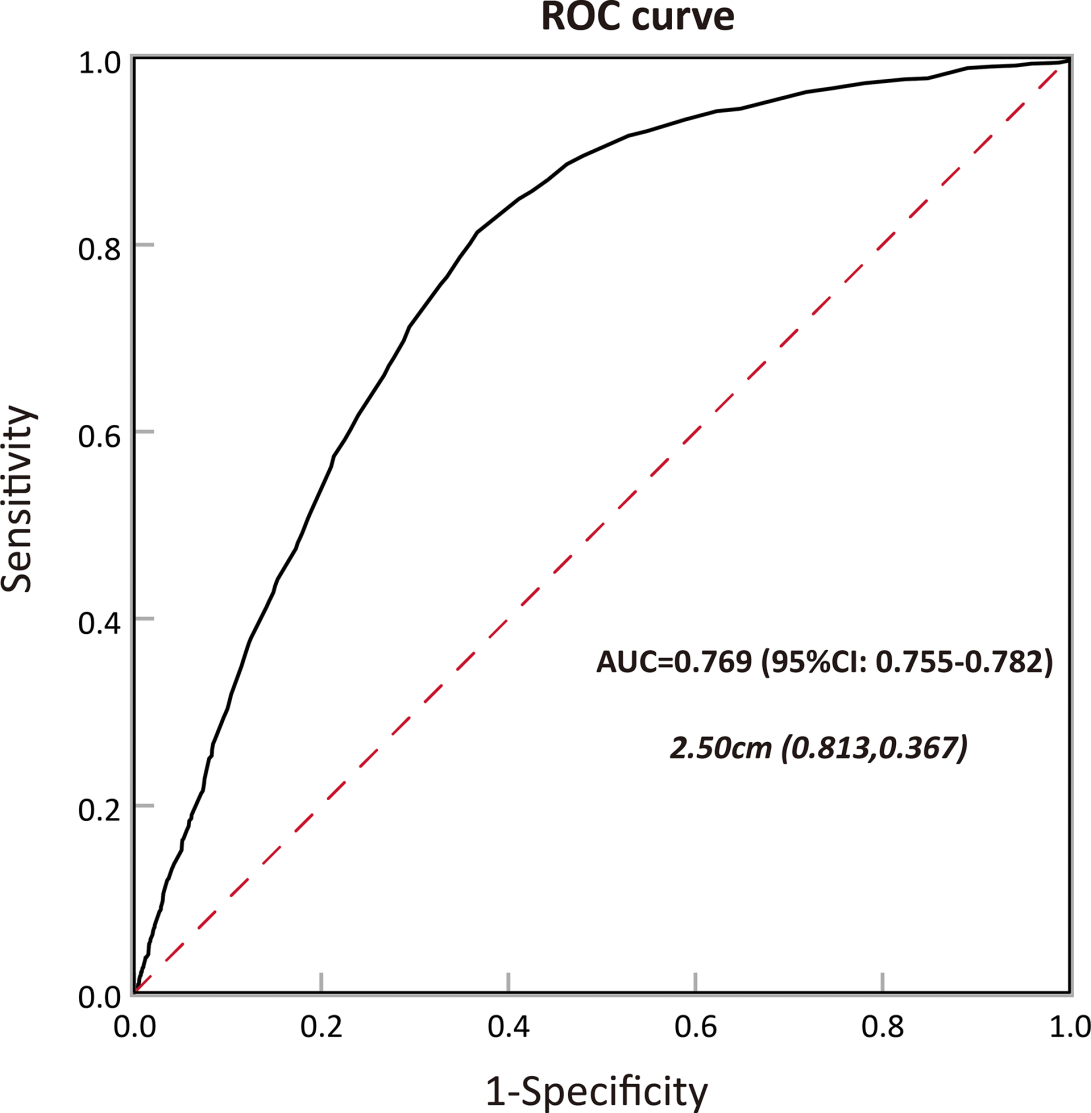
Calculation of the cutoff value for tumor size in predicting tumor aggressiveness among patients with pancreatic neuroendocrine neoplasms (PanNENs) by receiver operating characteristic (ROC) analysis and the area under the curve (AUC).

In order to determine the association between tumor size and aggressive behavior, the predictive ability of preoperative size in the subset of NF-PanNENs was evaluated using the ROC method. In our study, tumor aggressiveness was defined as poor tumor differentiation, lymph node involvement, liver involvement, and advanced tumor stage. In receiver operating characteristic curve analysis, tumor size resulted in an area under the curve (AUC) of 0.769 (95% CI, 0.755–0.782), showing a satisfactory ability to predict aggressiveness in patients with NF-PanNENs. The optimum tumor size cutoff value distinguishing tumor aggressiveness was 2.50 cm, resulting in 81.3% sensitivity and 63.3% specificity (**Figure 3**).

### Characteristics of NF-PanNETs ≤2 cm Between Observation and Surgery Cohorts

Among the 5,172 patients with PanNENs enrolled in the SEER database, 1,621 patients were diagnosed with NF-PanNETs according to the WHO classification, of whom 1,350 underwent surgery and 271 performed a conservative treatment. Baseline demographics and clinicopathologic features were displayed in **Table 2**. As shown in the table, age at diagnosis, the rates of lymph node involvement and liver involvement, as well as tumor stage were significantly different between these two cohorts. In the surgery cohort, patients were more frequently presented with lymph node involvement and loco-regional disease.

**Table 2:** Comparison of baseline characteristics before and after PSM in patients with NF-PanNETs ≤2 cm.

Variables	Before PSM			After PSM		
	Observation (*n* = 271)	Surgery (*n* = 1,350)	*p*-value	Observation (*n* = 259)	Surgery (*n* = 259)	*p*-value
Age			<0.001^*^			1.000
<65 years	128 (47.2%)	863 (63.9%)		122 (47.1%)	122 (47.1%)	
≥65 years	143 (52.8%)	487 (36.1%)		137 (52.9%)	137 (52.9%)	
Gender			0.081			0.429
Male	144 (53.1%)	639 (47.3%)		139 (53.7%)	130 (50.2%)	
Female	127 (46.9%)	711 (52.7%)		120 (46.3%)	129 (49.8%)	
Ethnicity			0.184			1.000
White	216 (79.7%)	1,012 (75.0%)		208 (80.3%)	208 (80.3%)	
Black	26 (9.6%)	158 (11.7%)		22 (8.5%)	22 (8.5%)	
Other	29 (10.7%)	180 (13.3%)		29 (11.2%)	29 (11.2%)	
Marital status			0.579			1.000
Married	167 (61.6%)	856 (63.4%)		160 (61.8%)	160 (61.8%)	
Other	104 (38.4%)	494 (36.6%)		99 (38.2%)	99 (38.2%)	
Tumor grade			0.863			0.537
Well differentiated	245 (90.4%)	1,225 (90.7%)		234 (90.3%)	238 (91.9%)	
Moderately differentiated	26 (9.6%)	125 (9.3%)		25 (9.7%)	21 (8.1%)	
Tumor number			0.736			0.588
Single	240 (88.6%)	1,205 (89.3%)		230 (88.8%)	226 (87.3%)	
Multiple	31 (11.4%)	145 (10.7%)		29 (11.2%)	33 (12.7%)	
Tumor location			0.071			1.000
Head	65 (24.0%)	320 (23.7%)		59 (22.8%)	59 (22.8%)	
Body/tail	150 (55.4%)	824 (61.0%)		148 (57.1%)	148 (57.1%)	
Other	56 (20.6%)	206 (15.3%)		52 (20.1%)	52 (20.1%)	
Lymph node involvement			0.002^*^			1.000
Yes	8 (3.0%)	109 (8.1%)		5 (1.9%)	5 (1.9%)	
No	263 (97.0%)	1,241 (91.9%)		254 (98.1%)	254 (98.1%)	
Liver involvement			<0.001^*^			1.000
Yes	19 (7.0%)	13 (1.0%)		9 (3.4%)	9 (3.4%)	
No	250 (92.3%)	1,325 (98.1%)		249 (96.1%)	249 (96.1%)	
Unknown	2 (0.7%)	12 (0.9%)		1 (0.4%)	1 (0.4%)	
Tumor stage			<0.001^*^			1.000
Localized	242 (89.3%)	1,165 (86.3%)		242 (93.4%)	242 (93.4%)	
Regional	8 (3.0%)	158 (11.7%)		7 (2.7%)	7 (2.7%)	
Distant	21 (7.7%)	48 (3.0%)		10 (3.9%)	10 (3.9%)	

PSM, propensity score matching; NF-PanNETs, nonfunctional pancreatic neuroendocrine tumors. ^*^Significance.

### Comparison of Survival Outcomes After Propensity Score Matching

**Figure 4:**
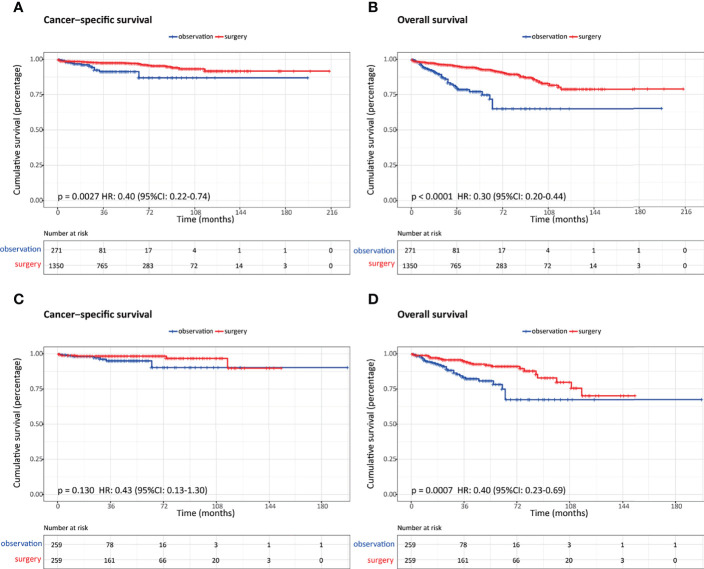
Comparison of survival outcomes in patients with nonfunctional pancreatic neuroendocrine neoplasms (NF-PanNENs) ≤2 cm who underwent observation and surgery before and after propensity score matching (PSM). **(A)** Cancer-specific survival before PSM. **(B)** Overall survival before PSM. **(C)** Cancer-specific survival after PSM. **(D)** Overall survival after PSM.

After PSM, 259 patients were matched in each cohort, and the baseline characteristics were well-balanced (**Table 2**). With regard to survival results, the overall survival was significantly better in patients who underwent surgery regardless of the propensity score analysis. While the cancer-specific survival was comparable between these two groups after propensity score matching (**Figure 4**.)

### Factors Associated With Patients With NF-PanNENs Who Underwent Surgery

On univariate logistic regression analysis, age at diagnosis, year of diagnosis, tumor size, functional status, lymph node status, liver involvement, and tumor stage were associated with patients who were more likely to receive surgical treatment. In multivariate analysis, diagnosis at early year, age (<65 years), size 1–2 cm, functional tumors, lymph node involvement, and liver involvement were significant predictors for patients treated with surgery (**Table 3**).

**Table 3:** Factors associated with patients with PanNENs ≤2 cm who underwent surgery in the SEER database.

Variables	Univariate analysis		Multivariate analysis	
	OR (95% CI)	*p*-value	OR (95% CI)	*p-*value
Age				
<65 years	Ref		Ref	
≥65 years	0.50 (0.39, 0.65)	<0.001^*^	0.51 (0.39, 0.67)	<0.001^*^
Gender				
Male	Ref			
Female	1.25 (0.97, 1.61)	0.092		
Ethnicity				
White	Ref			
Black	1.34 (0.87, 2.06)	0.182		
Other	1.35 (0.89, 2.04)	0.160		
Year of diagnosis				
Per year	0.84 (0.80, 0.89)	<0.001^*^	0.79 (0.74, 0.84)	<0.001^*^
Marital status				
Married	Ref			
Other	0.89 (0.69, 1.16)	0.401		
Tumor grade				
Well differentiated	Ref			
Moderately differentiated	1.22 (0.79, 1.87)	0.368		
Poorly differentiated	0.52 (0.24, 1.14)	0.101		
Tumor size				
≤1 cm	Ref		Ref	
1–2 cm	1.64 (1.25, 2.14)	<0.001^*^	1.60 (1.20, 2.12)	0.001^*^
Tumor number				
Single	Ref			
Multiple	0.93 (0.62, 1.39)	0.722		
Tumor location				
Head	Ref			
Body/tail	1.13 (0.83, 1.54)	0.441		
Other	0.72 (0.49, 1.05)	0.084		
Functional status				
Nonfunctional	Ref		Ref	
Functional	5.51 (1.73, 17.56)	0.004^*^	6.78 (2.01, 22.88)	0.002^*^
Lymph node involvement				
No	Ref		Ref	
Yes	2.68 (1.39, 5.17)	0.003^*^	4.43 (2.06, 9.52)	<0.001^*^
Liver involvement				
No	Ref		Ref	
Yes	0.16 (0.09, 0.31)	<0.001^*^	0.13 (0.08, 0.21)	<0.001^*^

PanNENs, pancreatic neuroendocrine neoplasms; OR, odds ratio; CI, confidence interval; Ref, reference. ^*^Significance.

### Analysis of Risk Factors for OS in Patients With PanNENs ≤2 cm

The estimated OS rates at 3, 5, and 10 years were 92.3%, 89.2%, and 75.6%, respectively, while the estimated CSS probabilities at 3, 5, and 10 years were 95.8%, 95.0%, and 89.6%, respectively. On multivariate analysis, age, gender, ethnicity, tumor grade, tumor number, and tumor stage were independent prognostic factors for OS in patients with small-sized PanNENs (**Table 4**).

**Table 4:** Factors associated with overall survival in patients with PanNENs ≤2 cm.

Variables	Univariate analysis		Multivariate analysis	
	HR (95% CI)	*p*-value	HR (95% CI)	*p*-value
Age				
<65 years	Ref		Ref	
≥65 years	3.50 (2.52, 4.84)	<0.001^*^	4.04 (2.86, 5.71)	<0.001^*^
Gender				
Male	Ref		Ref	
Female	0.69 (0.50, 0.94)	0.017^*^	0.65 (0.47, 0.90)	0.009^*^
Ethnicity				
White	Ref		Ref	
Black	1.68 (1.12, 2.53)	0.012^*^	2.26 (1.48, 3.44)	<0.001^*^
Other	0.64 (0.35, 1.16)	0.142	0.79 (0.43, 1.46)	0.459
Year of diagnosis				
Per year	0.95 (0.90, 1.01)	0.060		
Marital status				
Married	Ref			
Other	1.33 (0.98, 1.83)	0.072		
Tumor grade				
Well differentiated	Ref		Ref	
Moderately differentiated	1.43 (0.91, 2.25)	0.126	1.44 (0.91, 2.29)	0.124
Poorly differentiated	7.56 (4.58, 12.49)	<0.001^*^	6.10 (3.53, 10.53)	<0.001^*^
Tumor size				
≤1 cm	Ref			
1–2 cm	0.92 (0.65, 1.31)	0.652		
Tumor number				
Single	Ref		Ref	
Multiple	2.68 (1.91, 3.78)	<0.001^*^	2.33 (1.64, 3.31)	<0.001^*^
Tumor location				
Head	Ref			
Body/tail	1.00 (0.69, 1.46)	0.986		
Other	1.02 (0.63, 1.63)	0.952		
Functional status				
Nonfunctional	Ref			
Functional	1.25 (0.66, 2.38)	0.492		
Lymph node involvement				
No	Ref			
Yes	1.48 (0.94, 2.32)	0.093		
Liver involvement				
No	Ref		Ref	
Yes	5.75 (3.47, 9.54)	<0.001^*^	1.03 (0.35, 3.01)	0.961
Tumor stage				
Localized	Ref		Ref	
Regional	1.30 (0.82, 2.06)	0.257	0.98 (0.60, 1.61)	0.933
Distant	5.61 (3.70, 8.52)	<0.001^*^	4.60 (1.73, 12.23)	0.002^*^

PanNENs, pancreatic neuroendocrine neoplasms; HR, hazards ratio; CI, confidence interval; Ref, reference. ^*^Significance.

### Exploratory Analyses

**Figure 5:**
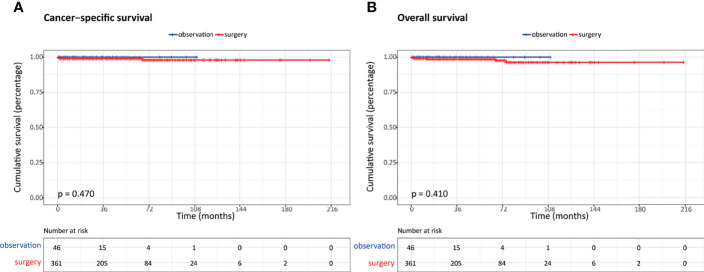
Comparison of survival outcomes in patients with highly selected nonfunctional pancreatic neuroendocrine neoplasms (NF-PanNENs) ≤2 cm between observation and surgery cohorts after propensity score matching (PSM). **(A)** Cancer-specific survival. **(B)** Overall survival.

In order to better define the appropriate indications for nonoperative management, we selected a cohort of patients based on the results of a multivariate survival analysis. Overall, a total of 407 patients were identified, of whom 46 underwent observation and 361 underwent aggressive surgery. In addition, the OS and CSS were comparable between these two groups (**Figure 5**).

## Discussion

Our study provides a comprehensive characterization of PanNENs and NF-PanNETs based on a large cohort of the population from the United States. Generally, the choice between observation and aggressive surgery should be on the basis of an accurate estimate of the malignant potential. However, our results indicate that using a 2-cm cutoff size alone to guide treatment decisions does not seem to be appropriate and safe. Surgery was found to be associated with survival advantages in patients with NF-PanNETs ≤2 cm compared to observation regardless of PSM analysis. Instead, patients with NF-PanNENs who were younger than 65 years old, of the female sex, white or other ethnicities rather than black, low-to-intermediate grade, with single tumor, and loco-regional stage were the suitable candidates for active surveillance.

The optimal management of NF-PanNETs less than or equal to 2 cm in size represented an unsolved clinical challenge in recent years, especially with the steadily increasing incidence of these incidentally discovered tumors. Lacking adequately powered studies investigating their clinical features and identifying the prognostic factors, indications for expectant observation in treating small tumors remain ambiguous and inconsistent. A preoperative tumor size had been proposed to predict the malignant potential and help in clinical decision-making (12, 13). Some consensus recommendations suggest that observation can be considered an option due to the clinically indolent and benign course of tumors less than or equal to 2 cm. Both the European Neuroendocrine Tumor Society (ENETS) Consensus Guidelines and NCCN guidelines offer an active surveillance strategy for patients with low-grade tumors measuring less than 2 cm in size, with a comprehensive assessment of the individual patient characteristics (14, 15). Lee et al. retrospectively reviewed 133 incidentally detected, small NF-PanNETs patients (77 nonoperative, 56 operatives), and they found that nonoperative treatment may be advocated as these tumors commonly showed minimal or no growth during follow-up (16). In a matched case-control study, Ssdot et al. analyzed the natural history of small (<3 cm), asymptomatic PanNETs and evaluated the efficacy of surgical resection versus observation. Among the patients who were initially observed, none of them developed distant metastases or died, with a median follow-up of 44 months (17). Similarly, Barenboim et al. identified 44 small asymptomatic NF-PanNETs treated with expectant observation between 2001 and 2018 and reported that no patients presented with regional or systemic disease progression or cancer-related death after a follow-up of 52.8 months. Considering the potential risks of preoperative morbidity and mortality, a strategy of conservative management seemed to be acceptable in selected patients (18). A systematic review including 5 retrospective literatures with 540 asymptomatic, small NF-PanNENs was conducted to evaluate the outcome between active surveillance with surgery. During the follow-up, the observation group did not occur disease-related deaths; therefore, they concluded that expectant management may be a reasonable alternative to aggressive surgery in highly selected patients (19). However, other studies have questioned the safety and feasibility of a conservative strategy and demonstrated that NF-PanNETs were associated with small but measurable malignant potential and aggressive surgical resection could provide long-term survival benefits (11). Gratian et al. found that 3 of 56 NF-PanNETs with tumors less than 2 cm developed metastatic disease and 2 of them died. In addition, tumor size was not related to distant metastasis or survival outcomes, which implied that it should not be used as an indication for treatment decisions (20). In a retrospective study including 3,243 cases with early-stage PanNETs ≤2 cm selected from the National Cancer Database, Chivukula et al. demonstrated a survival benefit of surgical resection for tumors 1 to 2 cm in size (21). Overall, the dilemma in managing patients with NF-PNETs ≤2 cm results from the benefits of surgery, which need to be weighed against the risks of possible disease progression, surgery-related morbidity, and comorbidities.

Generally, tumor differentiation based on the WHO classification and the AJCC staging system were regarded as two main determinants in the selection of optimal management for patients with PanNETs. While both of these two elements were not easily obtained before surgery, the preoperatively available clinical variable, tumor size, was frequently used to predict the tumor metastatic progression and aid clinical decision-making. However, as NF-PanNETs are a heterogeneous group of entities that exhibit a broad spectrum of biological behavior, there is no clear cutoff for benign disease. The current study demonstrates that tumor size alone cannot differentiate whether patients with NF-PNETs less than 2 cm are the appropriate candidates for an expectant observation, and other preoperatively available clinical features need to be taken into account as well, such as individual patient characteristics, comorbidities, and other risk factors for survival. In our study, a number of clinicopathological variables were identified to be associated with overall survival in NF-PanNENs ≤2 cm, including age, gender, ethnicity, tumor grade, tumor number, and tumor stage. Therefore, when considering the clinical factors that may inform the decision to perform observation or surgical resection, tumor size and these risk factors should act as a marker for the clinicians.

Our study had several limitations. The inherent biases with a retrospective design could not be completely eliminated even though we used propensity score matching. Secondly, the lack of important data in the SEER database may fail to incorporate some recognized prognostic parameters, such as the Ki-67 index and surgery-related complications. Last, the small size of patients after propensity score matching may therefore limit the generalization of the results.

In conclusion, expectant observation of small NF-PanNETs may be a reasonable alternative to aggressive surgical resection in highly selected patients. Also, the decision to observe versus surgery should not only be based on tumor size alone but also take into account other important clinicopathological factors. Further prospective multicentric studies and robust data are required to validate the benefit of this conservative policy.

## Data Availability Statement

Publicly available datasets were analyzed in this study. These data can be found here: https://seer.cancer.gov/.

## Author Contributions

GS contributed to the conception and designed the study. ZY drafted the manuscript. DZ conducted the statistical analysis. All authors listed have made a substantial, direct, and intellectual contribution to the work and approved it for publication.

## Conflict of Interest

The authors declare that the research was conducted in the absence of any commercial or financial relationships that could be construed as a potential conflict of interest.

## Publisher’s Note

All claims expressed in this article are solely those of the authors and do not necessarily represent those of their affiliated organizations, or those of the publisher, the editors and the reviewers. Any product that may be evaluated in this article, or claim that may be made by its manufacturer, is not guaranteed or endorsed by the publisher.
